# Case Report: Somatic *NF2* mutation in a vestibular schwannoma arising in a patient with neurofibromatosis type 1

**DOI:** 10.3389/fonc.2026.1769591

**Published:** 2026-02-26

**Authors:** Misa Shogaku, Hiroshi Yamada, Seiji Yamada, Ryota Fujinami, Shigeki Yamada, Motoki Tanikawa, Yusuke Okuno, Mitsuhito Mase

**Affiliations:** 1 Department of Neurosurgery, Nagoya City University Graduate School of Medical Sciences, Nagoya, Japan; 2 Division of Analytical Pathology, Oncology Innovation Center, Research Promotion Headquarters, Fujita Health University School of Medicine, Toyoake, Japan; 3 Department of Virology, Nagoya City University Graduate School of Medical Sciences, Nagoya, Japan

**Keywords:** germline mutation, neurofibromatosis type 1, neuro-oncology, somatic mutation, vestibular schwannoma, whole-exome sequencing

## Abstract

**Background:**

Neurofibromatosis type 1 (NF1) and vestibular schwannoma are genetically and clinically distinct entities, with vestibular schwannomas classically associated with neurofibromatosis type 2. The occurrence of a vestibular schwannoma in a patient with NF1 is rare, and its underlying molecular mechanism remains unclear.

**Case presentation:**

We report a 51-year-old man clinically diagnosed with neurofibromatosis type 1 who developed a unilateral vestibular schwannoma presenting with progressive hearing loss and neurological symptoms. Histopathological examination following surgical resection confirmed the diagnosis of vestibular schwannoma, and genetic analyses were subsequently performed on the schwannoma, a cutaneous neurofibroma, and peripheral blood from the same patient.

**Genetic findings:**

Whole-exome sequencing revealed a pathogenic germline NF1 mutation shared across all analyzed samples. In contrast, the vestibular schwannoma harbored a somatic NF2 mutation accompanied by loss of chromosome 22, while these alterations were absent in the neurofibroma and blood samples.

**Conclusion:**

This case demonstrates that a vestibular schwannoma arising in a patient with neurofibromatosis type 1 can be driven by secondary somatic NF2 alterations accompanied by loss of chromosome 22. Comprehensive multi-tissue genetic analysis enabled direct distinction between germline and tumor-specific events, highlighting the critical role of tumor-specific somatic alterations beyond the germline background.

## Introduction

Neurofibromatosis type 1 (NF1) is an autosomal dominant neurocutaneous disorder characterized by a range of clinical manifestations, including multiple neurofibromas, café-au-lait macules, inguinal freckling, optic gliomas, Lisch nodules, and skeletal abnormalities. The NF1 gene, located on chromosome 17q11.2, encodes the protein neurofibromin. Loss of neurofibromin leads to hyperactivation of the Ras/MAPK and mechanistic target of rapamycin (mTOR) signaling pathways, thereby contributing to tumorigenesis (1). Approximately 50% of NF1 cases are inherited, while the remainder arise *de novo*, with no family history (2). Clinical presentations vary widely and are likely influenced by epigenetic modifications (1). Neurofibromas develop in about 60% of NF1 patients and may occur either cutaneously or internally. Plexiform neurofibromas, which are pathognomonic for NF1, arise internally and may undergo malignant transformation into peripheral nerve sheath tumors (MPNSTs), with a lifetime risk of estimated at 8–13% (1).

Neurofibromatosis type 2 (NF2) is another autosomal dominant disorder, characterized by vestibular schwannomas, spinal schwannomas, meningiomas, and ependymomas, and juvenile cataracts. Vestibular schwannomas are typically solitary tumors; however, approximately 4% to 6% are associated with NF2 (3). The NF2 gene, located on chromosome 22q12.2, encodes the tumor suppressor protein merlin, which influences several key signaling pathways, including PI3 kinase(PI3K)/Akt, Raf/MEK/ERK, and mTOR. Mutations in the merlin gene are identified in approximately 93% of patients with clinical evidence of NF2 and a positive family history. However, more than half of the NF2 cases occur *de novo* in individuals without any family history (1).

By contrast, NF1 and NF2 are distinct autosomal dominant disorders characterized by different clinical and genetic features. NF1 is primarily associated with cutaneous neurofibromas and Ras pathway dysregulation, while NF2 is typified by vestibular schwannomas due to merlin deficiency. Although historically grouped under the umbrella of “neurofibromatosis,” these two conditions rarely present concurrently in a single patient.

The coincidental occurrence of NF1 and NF2-related manifestations raises intriguing questions about potential genetic crosstalk or sequential mutational events that remain poorly characterized. To date, reports of such overlap remain exceedingly rare.

Here, we describe a unique case of a patient fulfilling clinical criteria for NF1 who concurrently developed a vestibular schwannoma, with genetic analysis revealing both a germline *NF1* mutation and a somatic *NF2* mutation in tumor tissue.

## Case presentation

A 51-year-old man presented with left-sided hearing loss and dysarthria. He also reported a progressive gait disturbance that had gradually worsened over several months. Neurological examination revealed left-sided sensorineural hearing loss (4-frequency pure-tone average of 62.5 dB), facial nerve palsy, and dysmetria with associated coordination disturbances.

The patient had a long-standing history of cutaneous abnormalities, including multiple pigmented macules and subcutaneous nodules, which had not previously been evaluated. Dermatological consultation identified these lesions as café-au-lait spots and neurofibromas, leading to a clinical diagnosis of NF1. The patient also reported a family history of similar skin findings in his father, although no formal diagnosis or treatment had been documented. Other diagnostic criteria for NF1, such as axillary freckling, Lisch nodules, or skeletal dysplasia, were absent.

**Figure 1:**
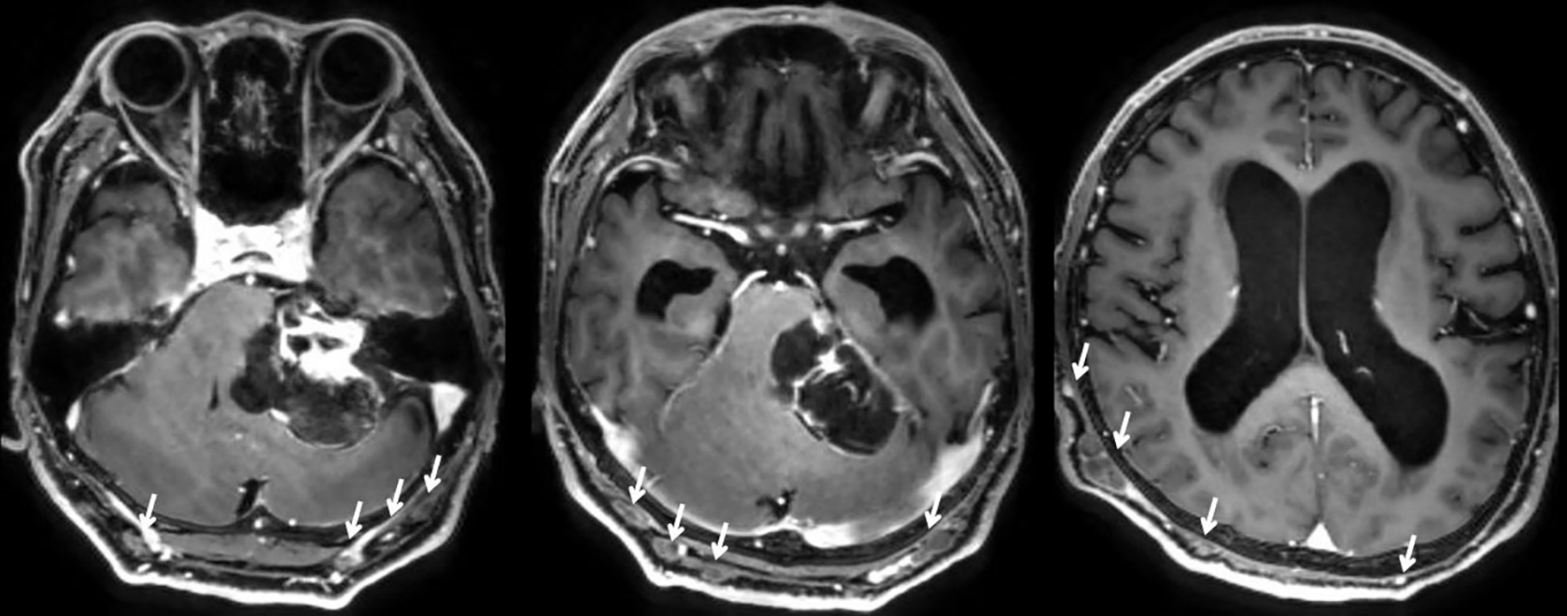
Preoperative axial contrast enhanced T1-weighted MRI demonstrating a mass lesion in the left cerebellopontine angle. The lesion shows well-enhanced solid components and cystic components, causing significant compression of the cerebellum and brain stem, along with associated hydrocephalus. Additionally, multiple subcutaneous mass lesions are observed (white arrows).

Contrast-enhanced magnetic resonance imaging (MRI) of the head revealed a heterogeneously enhancing mass, approximately 5 cm in diameter, located in the left cerebellopontine (CP) angle (**Figure 1**). The lesion exerted significant compression on the cerebellum and brainstem, caused enlargement of the internal auditory canal, and led to obstructive hydrocephalus. The tumor exhibited both solid components with intense gadolinium enhancement and cystic areas. Notably, multiple subcutaneous masses were also visualized on the same imaging studies.

**Figure 2:**
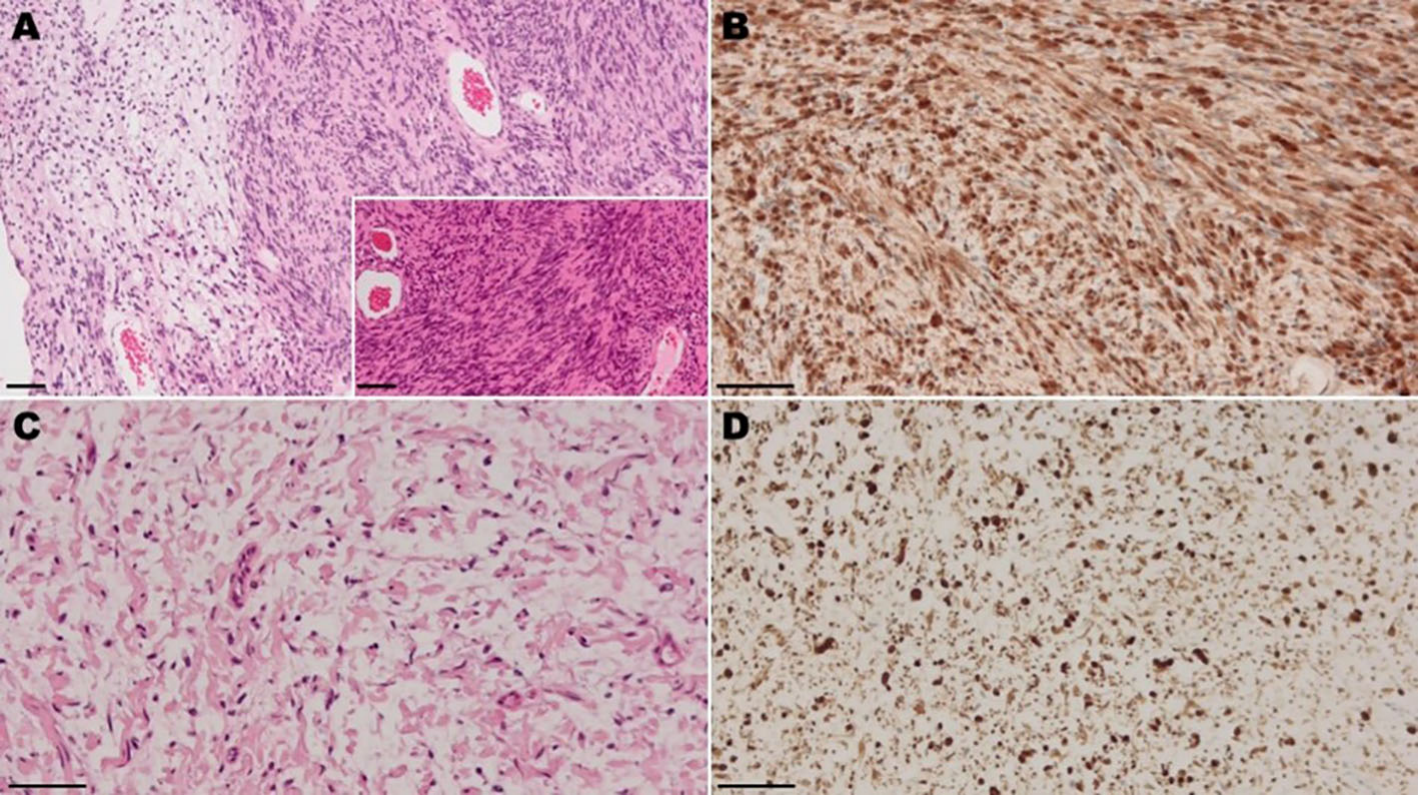
Light micrographs of the surgical specimen **(A, B)** Intracranial tumor. **(A)** Hematoxylin and eosin (H&E) staining. The tumor shows biphasic pattern with compact area (Antoni A tissue; right) adjacent to loosely arranged area (Antoni B tissue; left). The tumor of Antoni A tissue is composed of spindle cells showing occasional nuclear palisading, known as Verocay bodies (inset). **(B)** Immunohistochemically, the tumor cells are diffusely positive for S-100 protein. **(C, D)** Subcutaneous tumor. **(C)** H&E staining. The tumor shows proliferation of thin wavy cells immersing in a myxoid stroma. **(D)** Immunohistochemically, the tumor cells are diffusely positive for S-100 protein.

The CP angle mass was subtotally resected via a lateral suboccipital craniotomy. A subcutaneous mass lesion located near the surgical incision was also excised. The postoperative course was uneventful, with improvement in neurological symptoms except for complete deafness on the operated side, and follow-up MRI has shown no evidence of tumor regrowth to date. Histopathological examination confirmed the CP angle lesion as a schwannoma, showing characteristic Antoni A and B patterns (**Figure 2A**), with diffuse S-100 positivity (**Figure 2B**). The schwannoma showed a low Ki-67 labeling index of approximately 1%. In contrast, the subcutaneous tumor was consistent with a neurofibroma, showing thin wavy cells in a myxoid stroma (**Figure 2C**) and diffuse S-100 positivity (**Figure 2D**).

## Methods

### Patient

The patient provided informed consent, and the study was approved by the Institutional Review Board (IRB) of Nagoya City University (IRB number: 70-19-0003).

To investigate the underlying genetic etiology, we performed whole-exome sequencing on peripheral blood leukocytes, a subcutaneous neurofibroma, and a vestibular schwannoma, essentially as previously described (4).

### Exome sequencing

Briefly, genomic DNA was extracted from fresh tumor tissues and blood samples using the QIAamp DNA Blood Mini Kit (QIAGEN, Hilden, Germany), following the manufacturer’s instructions. Exome capture was performed using Agilent SureSelect Human All Exon v6, and sequencing was conducted on the Illumina NovaSeq platform (Illumina, San Diego, CA, USA). Germline and somatic point mutations, using the peripheral blood as the reference, were identified using VarScan2 (5) and annotated with ANNOVAR (6). Copy number alterations were detected by comparing sequencing coverage between the sample of interest and 12 unrelated germline samples. Loss of heterozygosity and uniparental disomy were evaluated based on the variant allele frequencies of common single nucleotide polymorphisms (SNP) (minor allele frequency >1%) to identify runs of homozygosity (7).

## Results

Our whole-exome sequencing analysis of peripheral blood, a subcutaneous neurofibroma, and a vestibular schwannoma identified a germline *NF1* mutation in all three samples, as well as eight somatic genetic alterations in the schwannoma sample (**Table 1**).

**Table 1:** Mutations identified in this study.

Tissue	Mutation	Zygosity
Peripheral blood	*NF1* c.2351G>T, p.Trp784Leu	Heterozygous
Neurofibroma	*NF1* c.2351G>T, p.Trp784Leu	Heterozygous
Vestibular schwannoma	*NF1* c.2351G>T, p.Trp784Leu	Heterozygous
Vestibular schwannoma	*STAT3* c.1889-2A>T, splice site (exon 21)	Heterozygous
Vestibular schwannoma	*TRIM72* c.884C>T, p.Pro295Leu	Heterozygous
Vestibular schwannoma	*IL23R* c.1783G>T, p.Val595Phe	Heterozygous
Vestibular schwannoma	*RNF180* c.1421G>A, p.Arg474Gln	Heterozygous
Vestibular schwannoma	*NF2* c.1282delC, p.Gln428Argfs*11	Hemizygous
Vestibular schwannoma	*SNTG2* c.295G>A, p.Val99Ile	Heterozygous
Vestibular schwannoma	*ATP13A4* c.323C>T, p.Pro108Leu	Heterozygous
Vestibular schwannoma	chr22 loss	Heterozygous

The germline *NF1* mutation was a missense variant, c.2351G>T (p.Trp784Leu), which is not listed in either SNP databases or pathogenic mutation databases. However, the 784th amino acid residue is evolutionarily conserved and located within a functionally important domain of neurofibromin, where pathogenic variants have been frequently reported (8, 9), and we interpreted this variant as pathogenic in accordance with the ACMG guidelines (criteria:PM1, PM2, and PP4) (10).

Somatic alterations in the schwannoma included an *NF2* frameshift mutation (c.1282delC, p.Gln428Argfs*11) and a deletion of the entire chromosome 22, resulting in the loss of the wild-type *NF2* allele. Consequently, the *NF2* frameshift mutation was present in a hemizygous state. The remaining six somatic point mutations were considered passenger mutations, as they were not found in somatic mutation databases such as COSMIC (http://cancer.sanger.ac.uk/cancergenome/projects/cosmic/). No copy number loss or uniparental disomy events affecting *NF1* were detected in the vestibular schwannoma.

No somatic mutations were identified in the subcutaneous neurofibroma.

## Discussion

The development of schwannomas is a hallmark of NF2, a genetically distinct disorder from NF1. Schwannomas are rare in NF1, with only a limited number of cases reported in the literature (2, 3, 11–17). Nevertheless, patients with NF1 are predisposed to a broad spectrum of tumors, as demonstrated by previous large population-based cohort studies (18). Non-malignant tumors commonly associated with NF1 include neurofibromas, Lisch nodules, optic pathway gliomas, and other low-grade central nervous system gliomas. Malignant neoplasms include malignant peripheral nerve sheath tumors (MPNSTs), juvenile myelomonocytic leukemia (JMML), pheochromocytoma, rhabdomyosarcoma, and glioblastoma multiforme (19). However, vestibular schwannomas in patients with NF1 are exceedingly rare. To the best of our knowledge, the only previously reported case involving genetic analysis was by A. Huq et al., who described a unilateral vestibular schwannoma harboring a somatic *NF2* mutation—findings that align with those observed in the present case (2).

Most unilateral vestibular schwannomas arise sporadically and are not associated with germline *NF2* mutations (20). However, somatic *NF2* mutations have been frequently identified in sporadic vestibular schwannomas, with reported prevalence rates ranging from 15% to 100% (21–28). Furthermore, approximately 50% of NF2 cases are *de novo*, and over 30% exhibit somatic mosaicism (29, 30). Mohyuddin et al. suggested that the presence of a unilateral vestibular schwannoma in a young individual should prompt evaluation for mosaic *NF2* mutations (31). In previously reported NF1 cases with co-occurring schwannomas that underwent genetic analysis, *NF2* mutations were identified in the tumor tissue but not in peripheral blood samples, suggesting a somatic origin. However, the possibility of mosaicism could not be completely excluded (2, 15). Yoo et al. described a unique case in which a patient with a germline *NF1* mutation exhibited a clinical phenotype more consistent with NF2 than with NF1 (15). They hypothesized that an *NF1* splicing mutation might influence tumor formation in tissues harboring *NF2* mutations, although the precise mechanism remains unclear.

Taken together, these observations raise the question of why schwannomas, typically associated with NF2, occasionally occur in patients with NF1.To explore this, it is important to consider the biological consequences of NF1 loss at the cellular level.

In patients with NF1, a pathogenic variant is present in one allele of the *NF1* gene in all somatic cells, resulting in a baseline susceptibility to tumor development. When the remaining wild-type allele is lost, there is a complete loss of neurofibromin function, which further enhances tumorigenic potential. Neurofibromin negatively regulates the Ras signaling pathway by accelerating the conversion of active Ras-GTP to inactive Ras-GDP. Loss of neurofibromin leads to elevated Ras-GTP levels, which in turn enhance signaling through Raf kinase and activate the downstream MEK and MAPK (Erk1/2) pathways, ultimately promoting cell proliferation (32).

One possible explanation is that germline *NF1* mutations may predispose cells to acquiring somatic *NF2* mutations. *NF1* mutations drive Ras pathway hyperactivation, creating a cellular environment characterized by excessive proliferative signaling and increased genomic instability, consistent with previous observations of elevated Ras-GTP levels and the requirement for additional genetic events in NF1-deficient cells (33).

In the present case, genetic analyses of peripheral blood, vestibular schwannoma, and a subcutaneous neurofibroma revealed a germline *NF1* mutation and a somatic *NF2* mutation restricted to the schwannoma. These findings support a dual-pathogenic mechanism involving both *NF1* and *NF2*. A permissive tumorigenic background associated with a germline NF1 mutation, together with a somatic NF2 mutation in Schwann cells, may provide a plausible biological explanation for schwannoma development. The possibility of mosaicism cannot be entirely excluded; nevertheless, it was considered unlikely given the unilateral vestibular schwannoma, absence of other NF2-associated features, and negative family history. Ongoing clinical follow-up is warranted to monitor for the potential development of bilateral vestibular schwannomas or other NF2-associated tumors.

## Conclusion

This case demonstrates that vestibular schwannoma arising in a patient with neurofibromatosis type 1 can be associated with secondary somatic *NF2* alterations accompanied by loss of chromosome 22. By performing comprehensive genetic analyses of three distinct tissues—peripheral blood, a cutaneous neurofibroma, and the vestibular schwannoma—from the same patient, we were able to directly distinguish germline and tumor-specific genetic events, revealing that *NF2* alterations were restricted to the vestibular schwannoma. These findings highlight the importance of tumor-specific somatic events, in addition to the germline background, in the development of NF2-related tumors in NF1 patients.

## Data Availability

The datasets presented in this article are not readily available because they contain potentially identifiable patient information and are restricted by the conditions of the institutional ethics committee approval. Requests to access the datasets should be directed to the corresponding author and will be considered in accordance with institutional policies and ethical regulations.
